# Revisiting telegony: offspring inherit an acquired characteristic of their mother's previous mate

**DOI:** 10.1111/ele.12373

**Published:** 2014-09-30

**Authors:** Angela J Crean, Anna M Kopps, Russell Bonduriansky, Dustin Marshall

**Affiliations:** Evolution and Ecology Research Centre and School of Biological, Earth and Environmental Sciences, University of New South WalesSydney, NSW, 2052, Australia

**Keywords:** Condition, diet, inheritance of acquired traits, non-genetic inheritance, paternal effect, plasticity, seminal proteins, telegony

## Abstract

Newly discovered non-genetic mechanisms break the link between genes and inheritance, thereby also raising the possibility that previous mating partners could influence traits in offspring sired by subsequent males that mate with the same female (‘telegony’). In the fly *Telostylinus angusticollis*, males transmit their environmentally acquired condition via paternal effects on offspring body size. We manipulated male condition, and mated females to two males in high or low condition in a fully crossed design. Although the second male sired a large majority of offspring, offspring body size was influenced by the condition of the first male. This effect was not observed when females were exposed to the first male without mating, implicating semen-mediated effects rather than female differential allocation based on pre-mating assessment of male quality. Our results reveal a novel type of transgenerational effect with potential implications for the evolution of reproductive strategies.

## Introduction

Recent advances in our understanding of inheritance have revealed that offspring-parent resemblance cannot be explained solely by the transmission of parental genes (see recent reviews: Danchin *et al*. 2011; Bonduriansky 2012). Accumulating evidence shows that a variety of inheritance mechanisms (including but not restricted to epigenetic inheritance) operate alongside Mendelian inheritance, such that both genetic and non-genetic sources of variation (and the interactions between them) can influence phenotypic variation and evolutionary outcomes. The recognition of non-genetic processes in the transmission of variation across generations necessitates a re-examination of phenomena excluded by classical genetics.

Before the advent of modern genetics, many biologists believed that a male can leave a mark on his mate's body, causing the female's subsequent offspring to resemble their mother's first mate, despite being sired by another male (Rabaud 1914; Ewart 1920). This hypothesised phenomenon, dubbed ‘telegony’ by August Weismann, was rejected in the early 20th century because it lacked unequivocal empirical support and was deemed incompatible with Mendelian genetics (Burkhardt 1979). However, recent discoveries have revealed the existence of molecular and physiological mechanisms that have the potential to mediate telegony (Liu 2011, 2013). Although classic discussions of telegony focused on effects carried over from one gestation to the next, similar mechanisms could enable males who do not sire any offspring to influence the development of future offspring sired by other males.

**Figure 1:**
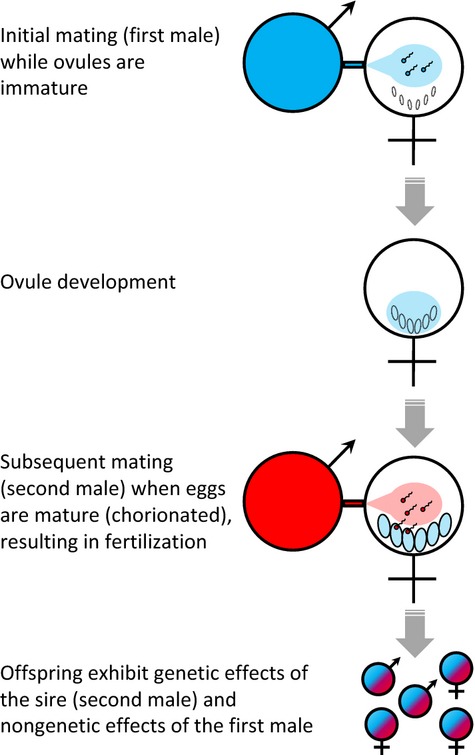
The hypothesised mechanism of telegony in *Telostylinus angusticollis*: From an initial mating (first male) that occurs while the ovules are immature and permeable to semen-borne molecules, the female receives seminal fluids that influence ovule development (shown in blue). A subsequent mating (second male), which occurs after ovule maturation, results in fertilisation, but is not expected to result in semen-mediated effects because mature (chorionated) eggs are largely impermeable to seminal products. The resulting offspring therefore exhibit a non-genetic influence of the phenotype of the first male (represented by blue colour), while also expressing alleles received from the second male (represented by red colour).

Potential mechanisms of telegony include penetration of maternal somatic cells by sperm, foetal genes in mother's blood, and the ability of RNA to program genome rearrangement (Liu 2011, 2013). In addition, males provide the female with a suite of proteins and other molecules in the seminal fluid (Avila *et al*. 2011), the concentration and composition of which can be altered by the male's environment (Wigby *et al*. 2009; Perry & Rowe 2010; Sirot *et al*. 2011). Seminal products are known to have complex effects on female fitness and behaviour (Gillott 2003; Wigby *et al*. 2009; Perry *et al*. 2013), and can influence offspring health via effects on the female reproductive tract (Bromfield 2014; Bromfield *et al*. 2014). Hence, we propose that the phenotype of a female's previous mate could potentially influence her future offspring, sired by other males, via the effects of seminal fluid on ovule development (Fig.1).

Previously, we have demonstrated in the neriid fly, *Telostylinus angusticollis*, that males reared on a nutrient-rich larval diet (high-condition fathers) produce larger offspring than males reared on a nutrient-poor larval diet (low-condition fathers) (Bonduriansky & Head 2007; Adler & Bonduriansky 2013). This paternal effect is especially interesting because there is no evidence of any conventional form of paternal provisioning or nuptial gift in this species: mean copulation duration is only 43 s, there is no external or internal spermatophore or mating plug, and mean ejaculate size is < 0.01% of male body volume (Bonduriansky & Head 2007; Bath *et al*. 2012). The effect of paternal condition on offspring size could be mediated by the transfer of condition-dependent accessory-gland products in the seminal fluid.

**Figure 2:**
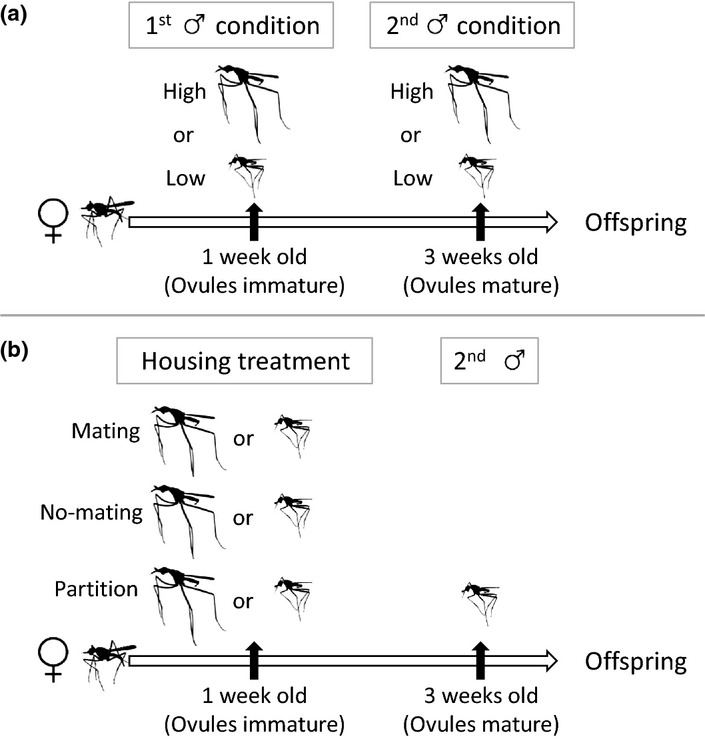
Experimental design. (a) Telegony experiment: Male condition was manipulated by rearing larvae on rich (high condition) or poor (low condition) larval diets. Females were mated to a male in high or low condition (‘first male’) 1 week after emergence (while their eggs were immature), and then remated to another male (‘second male’) 2 weeks later (when their eggs were mature) in a fully crossed design. Offspring from each mating combination were collected after the second mating. (b) Female differential allocation experiment: Females (1 week old) were either mated with (mating treatment), allowed to interact but not mate with (no-mating treatment), or housed adjacent to (partition treatment) a male in high or low condition. Two weeks later females were mated to a low condition male (second male) and offspring were collected.

To test for telegony in *T. angusticollis*, we manipulated male larval diet quality to generate variation in male condition, and mated recently eclosed females with a male in high- or low-condition (first male) to expose developing ovules to seminal fluid from these males. Two weeks later, after the females’ eggs matured, we re-mated each female with a new high- or low-condition male (second male) in a fully crossed design, and quantified phenotypic traits in offspring produced after this second mating (Fig.2a). Ovules are encased in a hard, largely impermeable chorion shell upon reaching their mature size, and then fertilised as they pass down the oviduct just before oviposition. Females store sperm in spermathecal ducts, but few females lay viable eggs 2 weeks after mating (AJC and RB, unpublished data).We therefore expected the second male to be the genetic sire of the offspring, and asked whether the first male can nonetheless influence offspring phenotype via non-genetic semen-mediated effects on the development of pre-chorionated ovules (Fig.1). As first male effects on offspring could also be mediated by female differential allocation of resources to developing ovules based on pre-mating assessment of male quality (Burley 1988; Sheldon 2000), we conducted a second experiment to verify the role of semen in mediating the first male effect, whereby recently eclosed females were either allowed to mate with a male in high or low condition or exposed to the male without mating (Fig.2b).

## Materials and methods

### Study species

*Telostylinus angusticollis* are polyandrous, forming mixed-sex mating aggregations on the trunks of beetle-damaged *Acacia longifolia* trees. As in all holometabolous insects, adult size and shape is fixed upon eclosion. Males reared on a rich larval diet develop exaggerated secondary sexual characters and are much larger than females, whereas males and females reared on a poor larval diet are similar in size and shape (Bonduriansky 2007; Sentinella *et al*. 2013). Large body size is advantageous in male–male contests for access to territories and females (Bonduriansky & Head 2007; Bath *et al*. 2012). Laboratory stocks of *T. angusticollis* were collected from Fred Hollows Reserve, Sydney, Australia (33.912°S, 151.248°E), and maintained in the laboratory as a large outbred population, supplemented annually with new wild-collected flies.

### Manipulation of condition

To obtain males in high and low condition, eggs collected from stock cages were transferred into containers of 50 eggs per 200 mL of fresh ‘rich’ or ‘poor’ larval medium, which represents *ad libitum* food for larvae (Bonduriansky & Head 2007). Rich larval medium contains 3-fold higher concentrations of protein and carbohydrates than poor larval medium (see Bonduriansky 2007 for details). Males raised on a rich larval diet were significantly larger than males raised on a poor larval diet (male thorax length mean ± SE: rich = 2.552 ± 0.046 mm, poor = 1.726 ± 0.017 mm; t_100_ = 17.111, *P *<* *0.001).

Larval containers were kept in a controlled-environment chamber set to an alternating light–dark 12–12 h cycle of 25/23°C and 50% humidity, and periodically misted with water. Upon eclosion, flies were separated by sex and larval diet treatment, and housed with *ad libitum* food (brown sugar and yeast) and water: poor females were discarded; rich females were housed individually in 250 mL containers; rich and poor males were housed in groups of 10 individuals in 2 L containers. Of females used as mothers in the experiment (*n *=* *26 per treatment combination), two died before the second mating (one low-high, one high-high treatment) and were excluded from analysis. Female body size did not differ among treatment groups (*F*_2,101_ = 0.702; *P *=* *0.498). No treatment males died during the experiment.

### Telegony experiment

Seven days after eclosion (while their ovules were still immature), females were paired with either a high-condition (rich larval diet) or low-condition (poor larval diet) male, and left to mate for 24 h. The males were then returned to their group cages and females left to mature in their individual cages. Females do not lay eggs unless given appropriate oviposition media, and therefore did not lay any eggs during this time. Two weeks after the initial mating (when their ovules were mature), females were paired to a second male for 24 h in a fully crossed design, resulting in four combinations of first and second male condition (high–high, high–low, low–high, low–low), and given oviposition medium to lay eggs (Fig.2a). To avoid cohort effects, the second male was drawn from the same set of males that had been used for the first mating, such that each male was used both as a first male and as a second male. We allowed 2 weeks between the first and second matings to allow females’ ovules to mature and minimise the prevalence of viable sperm from the first mating in female sperm-storage organs. Twenty randomly chosen eggs from each female were transferred into a container with 100 mL of poor larval medium. A subsample of eggs from each female (mean *n *=* *9.08, SD = 2.30, Table S1) was also photographed under a Leica MS5 stereoscope (Leica Microsystems, Wetzlar, Germany) fitted with a Leica DFC420 camera, and egg area was measured from images using Image J software (National Institutes of Health, Bethesda, Maryland, USA).

Larvae were left to develop in a controlled-environment chamber, as described above. Adult flies (offspring) were allowed to emerge into 2 L cages, with food and water provided *ad libitum*. The date of first emergence was recorded for each family (replicate), and after 10 days (when flies had ceased emerging) all adult offspring were counted and frozen (see Table S1 for sample size). In three replicates (one from each treatment combination except low–low) none of the eggs collected emerged as adults, so these replicates were excluded from analyses of offspring body size. Offspring (*n*_total_ = 1415) were later sexed and photographed in lateral view (after removing wings and legs) using the Leica MS5 stereoscope, and Image J was used to measure thorax length as an index of body size.

### Paternity analysis

All parental generation males and females were frozen immediately after mating/oviposition and their thorax lengths later measured as described above. DNA was extracted from the parents and a subsample of five offspring per family where possible (Table S1) from high–low and low–high treatment groups. DNA was extracted with a Gentra PureGene DNA extraction kit (Qiagen), and six microsatellite markers (Tangus 2, 8, 9, 10, 15, 20 (see Kopps *et al*. 2013); Table S2) were amplified as described in Kopps *et al*. (2013), run on an ABI 3730 DNA Analyser, and analysed with GeneMapper ver. 3.7 software (both Applied Biosystems, Foster City, CA, USA). We successfully genotyped 205 individual offspring, their 48 mothers and probable sires (second males), and 30 of 51 alternative potential first males (males from low–low and high–high treatments). Individuals were excluded from the data set if less than four microsatellite loci amplified successfully (25 offspring and 26 potential sires), or if offspring had any mismatches with their mother (7 individuals). The individual identity of the second male was known, and the genotypes of most potential alternative sires were present in the data set. Hence, we assessed paternity for the putative sire (second male) and potential alternative sires (first males) by non-exclusion (i.e. zero mismatching alleles with the second male after the maternal contribution was accounted for, see Supporting Information for further details) using the output table in Cervus (Marshall *et al*. 1998).

### Female differential allocation experiment

To determine whether the effect of the first male's condition on offspring body size was mediated by semen-borne factors or by female differential allocation based on pre-mating assessment of male quality, we performed a separate experiment in which females were either mated to the first male (mating treatment), allowed to interact but not mate with the first male (no-mating treatment), or housed adjacent to the first male with a mesh partition between the male and female (partition treatment) (Fig.2b). The mating treatment allowed semen transfer by both males (as in the telegony experiment), whereas the no-mating treatment prevented ejaculate transfer by the first male, thus allowing us to test for differential allocation based on pre-mating assessment. The partition treatment was intended to establish whether male chemosensory and (limited) visual cues were sufficient to allow for female assessment of male condition and differential allocation (if any).

In the mating treatment, week-old females were allowed to mate with a male in either high or low condition (*n*_high_ = 18, *n*_low_ = 18) over a period of 24 h, as described above. In the no-mating treatment, females were paired with a high-condition or low-condition male (*n*_high_ = 19, *n*_low_ = 19) for 24 h, but mating was prevented by gluing the male genitalia shut. Males were briefly immobilised by cooling, and a drop of medical glue (Leukosan Ultra High Viscosity Cyanoacrylate) was placed over the epandrium to seal the genitalia. Following recovery, males behaved normally, but were unable to achieve intromission or sperm transfer (AJC, pers. obs.). All males in this treatment were frozen after 36 h. In the partition treatment, females between 1 and 3 weeks of age were housed with a high- or low-condition male (*n*_high_ = 19, *n*_low_ = 18) on the opposite side of a mesh partition. At age 3 weeks, females from all treatments were provided with oviposition media to verify that no mating had occurred in the no-mating and partition treatments: as expected, only females in the mating treatment laid fertilised eggs that hatched into larvae. All females were then mated to a new male (all low-condition), and provided with fresh oviposition medium. From each female, 20 eggs were transferred to poor larval medium as described above, and adult offspring were counted, sexed and measured as described above (see Table S3 for sample sizes). Approximately 80% of females in this experiment produced adult offspring for measurement of adult body size (*n*_total_ = 994 adult offspring, Table S3).

### Data analysis

All data were analysed using JMP (version 10.0.0, SAS Institute Inc., Cary, NC, USA). From the telegony experiment, offspring body size (thorax length) and egg size (area) were analysed using linear mixed models fitted by restricted maximum likelihood (REML), with family included as a random effect, first male condition (high or low), second male condition (high or low) and offspring sex included as fixed, categorical effects, and maternal body size, second male body size and development time fitted as covariates. Models were re-fitted after removing non-significant interactions (always leaving all main effects) (Quinn & Keough 2002). To eliminate multicollinearity between body size and categorical predictors, male body size (thorax length) was normalised (mean = 0, SD = 1) within diet treatment, and offspring body size was normalised within sex. Egg-to-adult viability was measured as the number of offspring out of 20 eggs that emerged as adults, and developmental time was quantified as days between oviposition and first adult emergence. Both variables were analysed using a generalised linear model with Poisson distribution and log link function.

To verify that first male condition affected offspring sired by the second male, we re-analysed offspring body size using only those offspring that showed a genetic match to the second male. We fitted a linear mixed model as described above, except that instead of first and second male diet, we tested a treatment effect denoting first and second male's condition in crossover treatments (low–high vs. high–low). We also tested whether paternity varied with experimental treatment by fitting a generalised linear model (Poisson distribution, log link function), with the number of offspring per family sired by the second male as the dependant variable, treatment (high–low or low–high) included as a fixed, categorical effect, and maternal body size, second male body size and number of genotyped offspring per family fitted as covariates.

From the differential allocation experiment, offspring body size was analysed using a linear mixed model fitted by REML, with family fitted as a random effect, first male condition (high or low), housing treatment (mating, no-mating, partition) and offspring sex as fixed, categorical effects, and development time, maternal body size and second male body size fitted as covariates, along with all two- and three-way interactions. This model was re-fitted after eliminating covariates and interactions that were far from significance (*P *>* *0.2), and interaction contrasts (Quinn & Keough 2002) were carried out within this model by assigning coefficients (1 or −1) to specific combinations of treatment levels so as to define and test the interactions of interest between first male diet and the levels of the housing treatment (mating vs. no-mating; mating vs. partition). Female differential allocation based on pre-mating assessment would be manifested as an overall (main) effect of first male condition. Semen-mediated effects of first male condition would be manifested as a first male condition × housing treatment interaction, whereby high first male condition conferred increased offspring body size only when the first male was allowed to mate with the female.

## Results

### First and second male condition and offspring phenotype

**Figure 3:**
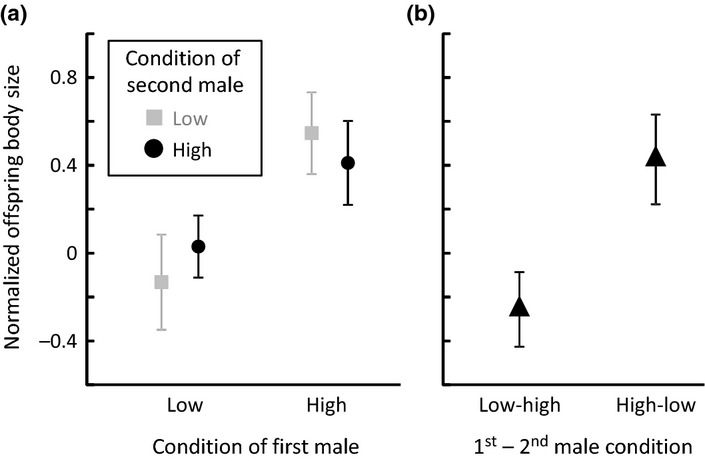
Offspring were larger when the first male was in high condition, independent of the condition of the second male. (a) Full data set and (b) reduced data set of genotyped offspring that were sired by the second male (low-high and high-low treatment groups only). Points show mean ± SE of family means.

We found that the adult body size of offspring was influenced positively by the condition of females’ initial mate (‘first male’): offspring were ∼ 0.5 SD larger when the female was initially mated to a high-condition male than when the female was initially mated to a low-condition male (Fig3a). However, offspring body size was not affected by the condition of females’ subsequent mate (‘second male’), or an interaction between first and second males’ condition (Table1a; Fig.3a; Table S4a; Fig. S1). Offspring body size was positively related to maternal body size, but not related to the body size of the second male (putative sire). There was also an association between offspring body size and developmental time (Table1a). However, developmental time was not affected by first or second male condition (Table S4d). The effect of first male condition on offspring body size was not mediated by egg size: larger females tended to produce larger eggs, but egg size was not affected by either the first or second male's condition (Table1b).

**Table 1:** Effects of first and second male condition (high vs. low) on offspring traits (full models including all non-significant interactions are shown in Table S4)

	Estimate	SE	d.f.	F	*P*
(a) offspring body size					
First male condition	0.229	0.090	1	**6.491**	**0.013**
Second male condition	0.006	0.088	1	0.004	0.948
Offspring sex	−0.014	0.015	1	0.794	0.373
Maternal size	0.213	0.095	1	**5.024**	**0.027**
Second male size	0.010	0.093	1	0.013	0.911
Developmental time	0.120	0.056	1	**4.505**	**0.037**
Family (random effect) = 0.700 ± 0.107					
(b) egg size					
First male condition	0.019	0.070	1	0.074	0.786
Second male condition	−0.096	0.070	1	1.938	0.167
Maternal size	0.004	0.002	1	3.723	0.057
Family (random effect) = 0.420 ± 0.070					

	Estimate	SE	d.f.	χ^2^	*P*
(c) Offspring egg-to-adult viability					
First male condition	−0.060	0.035	1	2.924	0.087
Second male condition	−0.025	0.035	1	0.524	0.470
Maternal size	−0.149	0.034	1	**18.667**	**<0.001**

Offspring body size (a) and egg size (b) were analysed using linear mixed models, with replicate (family) included as a random effect. The family variance component (proportion of total variance explained ± SE) is shown below the fixed effects. Offspring egg-to-adult viability (c) was analysed using a generalised linear model with Poisson distribution and log link function. Significant effects are highlighted in bold.

We observed a trend towards higher egg-to-adult viability of offspring when the first male was in low condition (mean viability = 76%) than when the first male was in high condition (mean viability = 65%). However, this effect was marginally non-significant when maternal body size was included in the model (Table1c, Fig. S2).

### Paternity analysis

As expected, paternity analysis based on microsatellite genotyping indicated that a large majority (87%) of offspring were sired by the second male (see Supporting Information, Fig. S3). In 35 families (offspring of the same mother) all genotyped offspring matched the second male, in five families there were no offspring that matched the second male, and in eight families some but not all offspring matched the second male. There was no difference between treatments in the proportion of offspring sired by the second male (χ^2^_1_ = 0.355, *P *=* *0.551), meaning that male condition did not affect relative siring success. Among offspring that were sired by the second male, we still found that offspring body size was influenced positively (by ∼ 0.7 SD) by the condition of the first male but not influenced by the condition of the second male (Table2, Fig.3b, Table S4e).

**Table 2:** Analysis of offspring adult body size, based on a reduced data set including only those offspring that showed a genetic match to the second male (full model including all non-significant interactions is shown in Table S4e)

	Estimate	SE	d.f.	F	*P*
Treatment	0.288	0.125	1	**5.307**	**0.027**
Offspring sex	−0.028	0.039	1	0.521	0.472
Maternal size	0.333	0.126	1	**6.970**	**0.012**
Family (random effect) = 0.718 ± 0.178					

‘Treatment’ denotes the condition of the first and second male (high-low vs. low-high). Effects were estimated in a linear mixed model, with family included as a random effect. Significant effects are highlighted in bold.

### Female differential allocation or semen-mediated effects?

**Figure 4:**
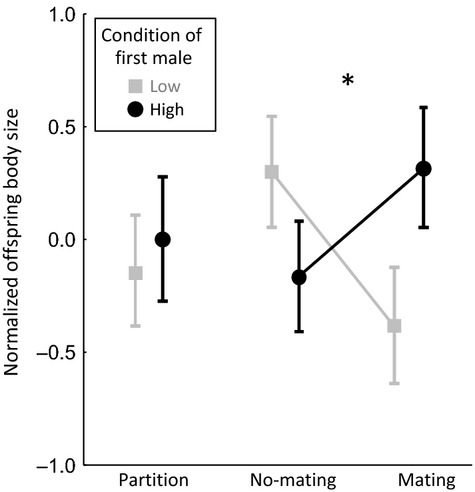
First male condition effects were mediated by semen rather than female differential allocation based on pre-mating assessment of male quality: first male condition influenced offspring body size when females were mated with the first male, but not when females were able to interact but not mate with the first male (significant interaction denoted by connecting lines and asterisk), or when females were separated from the first male by a partition. Points show least-squares means ± SE from the fitted model (Table S5).

In a separate experiment, where females were either allowed to mate with the first male or exposed to the first male without mating, an overall effect of first male condition was not observed (anova: first male condition: *F*_2,77.9_ = 0.16, *P *=* *0.854, Table S5). Thus, there was no evidence of differential allocation. Overall, the first male condition × housing treatment interaction was marginally non-significant (*F*_2,78.1_ = 2.52, *P *=* *0.087). However, when comparing the effect of first male condition in the mating vs. no-mating treatment groups, a significant interaction was observed (interaction contrast: *F*_1,77.6_ = 5.00, *P *=* *0.028; Fig.4): high first male condition resulted in increased offspring body size when the first male mated with the female, but not when the first male could interact but not mate with the female. This implicates semen-borne factors in mediating the effect of first-male condition on offspring body size. When comparing the effect of first male condition in the mating vs. partition treatment groups, the interaction was not significant (interaction contrast: *F*_1,78.5_ = 1. 04, *P *=* *0.310; Fig.4).

## Discussion

Our results show that it is possible for a male to transmit features of his phenotype via non-genetic semen-borne factors to his mate's subsequent offspring sired by another male. Offspring adult body size was influenced by the environmentally induced condition of their mother's first mate, with no effect of the condition of the second mate, which sired a large majority of the offspring. This effect persisted even when analysis was restricted to the second male's progeny. Because no first male effect was evident when females were exposed to the first male without mating, we conclude that the effect is mediated by semen-borne factors rather than by female differential allocation based on pre-mating assessment of male quality. Our study thus confirms the possibility of telegony via non-genetic (i.e. transgenerational plasticity) effects on non-offspring. The phenomenon reported here represents a new type of non-parental transgenerational effect and a novel source of variation in phenotype and fitness, with potential consequences for sexual coevolution.

The effect we report is ecologically plausible, and could have substantial consequences for fitness. Our manipulation of male condition mimics the effects of a subset of the natural variation in larval resource-patch quality (Bonduriansky 2007), and *T. angusticollis* females readily mate with multiple males in the wild (AJC and RB, unpublished observations). The effect we report could therefore occur in natural populations of this species. In previous studies (Bonduriansky & Head 2007; Adler & Bonduriansky 2013), we showed that *T. angusticollis* males reared on a rich larval diet produce offspring that are 0.5–1 SD larger than offspring produced by males reared on a poor larval diet, and that this paternal effect on offspring body size could have substantial consequences for offspring fitness. In particular, if the offspring experience a resource-poor larval patch, then daughters of rich-diet males would be expected to produce ∼ 27% more eggs per reproductive cycle, and sons of rich-diet males would likely enjoy a substantial advantage in male–male combat over access to territories and mates, relative to offspring of poor-diet males (Bonduriansky & Head 2007). In this study, we found that male condition can have an effect of similar magnitude on a female's future offspring sired by a different male. This novel transgenerational effect could therefore affect fitness of males and females in natural populations.

Our results implicate seminal fluid-borne factors in mediating the first male effect. Seminal fluid often makes up a substantial portion of the ejaculate, contains numerous substances that can alter female physiology and behaviour (Gillott 2003; Chapman 2008), and can be strategically allocated by the male (Wigby *et al*. 2009; Perry & Rowe 2010; Sirot *et al*. 2011). Most evidence of seminal protein effects comes from insects (reviewed in Avila *et al*. 2011; Perry *et al*. 2013). For example, in a ladybird beetle, *Adalia bipunctata*, male condition influences the concentration of non-sperm ejaculate components (Perry & Rowe 2010). Seminal proteins can influence egg development (Gillott 2003; Perry *et al*. 2013), and can be incorporated into eggs (Sirot *et al*. 2006), suggesting that seminal proteins have the potential to mediate non-genetic paternal effects. Evidence of semen-mediated effects on offspring development also exists in mammals (Chow *et al*. 2003; Robertson 2005; Wong *et al*. 2007; Bromfield *et al*. 2014): males that have their accessory glands removed sire offspring with abnormal physiological and behavioural phenotypes (reviewed in Bromfield *et al*. 2014). Our results suggest that seminal products can also mediate telegony.

As semen-mediated effects may only be possible when seminal products can penetrate developing ovules, such effects may be precluded when mating takes place after egg chorionation. We therefore predicted that the condition of the first male (with which females mated while their ovules were immature) would affect offspring development, but the condition of the second male (with which females mated when their eggs were already chorionated and ready to be laid) would not (Fig.1), and our results corroborate these predictions. [By the same token, we can reject an epigenetic (e.g. DNA methylation-based) mechanism: if the effect of male condition on offspring were mediated by epigenetic factors associated with sperm DNA, then the effect would be tied to fertilisation, predicting a second male effect but no first male effect in our experiment.” It is possible that effects of the second male's condition could have been detected in females’ subsequent broods (from ovules developing while exposed to semen of the second male). An alternative explanation for the lack of a second male effect on offspring traits is that second males were non-virgins and 2 weeks older than first males at the time of mating. Seminal fluid can change in composition as a male ages (Simmons *et al*. 2014), and females can become less responsive to the effects of semen with age (Fricke *et al*. 2013). This interpretation is unlikely to explain our results, however, because we have previously detected paternal effects when males were non-virgins, and when males and females were more than 3 weeks old (Adler & Bonduriansky 2013).

The transgenerational effect reported here is not a paternal effect because it affects non-offspring, but it can be regarded as a type of maternal effect whereby the phenotype of a female's previous mate influences her future offspring sired by another male. Interestingly, however, this effect occurs in a species in which the male transfers a tiny ejaculate that does not appear to contain any conventional form of nuptial gift (Bonduriansky & Head 2007). Hence, this effect could be taxonomically widespread.

Previous studies have suggested that semen from multiple males can interact to influence reproductive outcomes. For example, the presence of a previous male's seminal products can make the female insemination site a less hostile environment (Hodgson & Hosken 2006; Holman 2009), affect subsequent males’ sperm performance (den Boer *et al*. 2010; Simmons & Beveridge 2011; Locatello *et al*. 2013), and enhance female fecundity (Sirot *et al*. 2011). In addition, studies on the pseudoscorpion *Cordylochernes scorpioides* (Zeh & Zeh 2006) and the cricket *Teleogryllus oceanicus* (Garcia-Gonzalez & Simmons 2007) showed that a male can affect the viability of embryos sired by another male when both males mate in quick succession with the same female. Such interactions may also occur in multiple-paternity broods in mammals (Thonhauser *et al*. 2014). However, our study is the first to our knowledge to show that a male can affect the phenotype of offspring sired as much as 2 weeks later by another male, that this effect can occur even if the first male fails to achieve any fertilisations, and that such effects can extend to the adult phenotype of offspring.

Effects of a female's previous mate on a subsequent male's offspring could also come about via female differential allocation of resources to developing oocytes. Theory suggests that females may be selected to assess male quality, and preferentially allocate resources to the progeny of high-quality males (e.g. Burley 1988; Sheldon 2000; Kindsvater *et al*. 2013). If a female switches partners, assessment of the previous male could therefore affect the quantity of resources that a female invests in offspring sired by a subsequent male. However, we found no evidence of differential allocation based on pre-mating assessment of male quality in this study: male condition had positive effects on offspring body size when mating took place, but not when females were exposed to males without mating. We cannot exclude post-copulatory selection on the basis of chemical cues associated with the ejaculate (i.e. cryptic female choice; see Crean & Bonduriansky 2014). Interestingly, the apparent negative effect of first male condition on offspring body size in the no-mating treatment group (Fig.4) suggests that females suffer a cost (manifested in reduced offspring body size) from interacting with high-condition (large) males but, when mating takes place, this cost is offset by the positive effect on offspring body size of the semen transferred by these males. Semen-mediated effects on offspring quality may thus mitigate the harm to females resulting from pre-mating interactions with large males in this species.

The transgenerational effect we observed has the potential to play a unique role in evolution because it represents a distinct source of variation in fitness. The difference between this source of variation and both genetic inheritance and non-genetic parental effects is analogous to the difference between vertical and oblique transmission in cultural evolution. Whereas vertical transmission occurs from parent to offspring, oblique transmission occurs from an unrelated member of the parental generation, and theoretical studies have shown that oblique transmission can influence both the dynamics and equilibria of cultural evolution (e.g. see Cavalli-Sforza & Feldman 1981; Findlay *et al*. 1989; Gong 2010). For analogous reasons, in species lacking culture, oblique transmission (i.e. telegony) could influence evolutionary trajectories and equilibria – a possibility worth investigating in light of our findings.

Several predictions can be made. Because telegony decouples non-genetic transgenerational effects from fertilisation, it could have interesting consequences for both male and female mating strategies. Males could potentially exploit the reproductive investment of a female's previous mating partners, even in species lacking conventional forms of paternal provisioning. For example, in *T. angusticollis*, low-condition males may gain an offspring quality advantage by mating second to a high-condition male. Conversely, high-condition males might be selected to avoid mating with females that have previously mated with a low-condition partner. Such effects may select for the ability in males to discern (e.g. through chemical cues) the quality of a female's previous mating partners, providing a novel basis for male mate choice. Likewise, if non-genetic and genetic effects influence different components of offspring phenotype, females may be able to maximise offspring fitness via mate preferences that change over the course of the female ontogeny or reproductive cycle (Richard *et al*. 2005). For example, females may benefit by mating with males that optimise semen-dependent offspring traits while carrying immature ovules, but choosing males that optimise genetically determined offspring traits following ovule maturation (see Fig.1). Indeed, our results may account for observations of extreme choosiness in immature females, despite low probability of fertilisation as a result of strong last-male sperm precedence and/or lack of capacity for long-term sperm storage (Borgia 1981; Jones *et al*. 1998; Bonduriansky & Rowe 2003). Such female behaviour makes adaptive sense if male phenotype can influence offspring fitness without fertilisation, but not if females can simply ‘trade-up’ with sequential mate choice (Pitcher *et al*. 2003).

In summary, we show that adult body size of offspring can be influenced by the phenotype of a female's previous mate rather than the genetic sire in *Telostylinus angusticollis*. This novel transgenerational effect (an example of telegony) appears to be driven by the condition-dependent influence of male seminal fluid on the development of immature ovules. The potential for such effects exists in any taxon characterised by internal fertilisation and polyandry, and such effects could influence the evolution of reproductive strategies.
