# Supplementary material for: Revisiting telegony: offspring inherit an acquired characteristic of their mother's previous mate
Source: Ecol Lett. 2014 Sep 30;17(12):1545–52. doi: 10.1111/ele.12373 (PMC4282758; doi:10.1111/ele.12373)
Supplement: Supplementary file 1 [file ele0017-1545-sd1.doc]

**Supplementary Information**

*Paternity analysis*

As expected, microsatellite genotyping indicated that a large majority (178 out of 205) of successfully genotyped offspring from the high-low and low-high treatments were sired by the second male. Nonetheless, to assess whether the number of offspring matched to the second male using the non-exclusion method (zero mismatching alleles after the maternal contribution was accounted for) was higher than expected by chance, we programmed a randomisation test with 10000 iterations in MATLAB R2010a, and calculated both the number of individuals and the number of families with zero mismatches to a randomly assigned potential sire. In over 95% of iterations, ≤ 35 individuals had zero mismatches with a randomly assigned sire (Figure S3a). Thus, the number of offspring in our study matched to the second male (178) was approximately 5 times higher than what is expected if paternity is randomly drawn from all potential offspring-father pairs (Fisher’s exact p = 3.93 ×10^-49^). When examining offspring from the same mother (“families”), ≤ 3families were randomly assigned a single potential sire in over 95% of iterations. Thus, the number of families in our study that matched the second male in 100% of offspring (35) was over 10 times what is expected by chance (Fisher’s exact p = 8.47 ×10^-12^). Hence, we are confident that the non-exclusion method used to assign paternity did not overestimate the number of offspring matched to the second male by chance, but rather paternity assignment reflected our expectation that the majority of offspring would be sired by the second male (see Introduction and Methods).

**Table S1**. Sample sizes of telegony experiment, showing number of offspring of each sex that emerged as adults, and number of offspring from each family that were sequenced for paternity analysis.

| **family** | **1^st^ male  condition** | **2^nd^ male condition** | **# eggs** | **# female offspring** | **# male offspring** | **# offspring genotyped** |
| --- | --- | --- | --- | --- | --- | --- |
| ALL | low | low | 9 | 9 | 11 | - |
| BLL | low | low | 10 | 9 | 9 | - |
| CLL | low | low | 10 | 11 | 7 | - |
| DLL | low | low | 8 | 9 | 10 | - |
| ELL | low | low | 10 | 4 | 7 | - |
| FLL | low | low | 10 | 6 | 12 | - |
| GLL | low | low | 8 | 10 | 9 | - |
| HLL | low | low | 10 | 10 | 9 | - |
| ILL | low | low | 10 | 11 | 9 | - |
| JLL | low | low | 9 | 12 | 8 | - |
| KLL | low | low | 11 | 5 | 6 | - |
| LLL | low | low | 10 | 9 | 11 | - |
| MLL | low | low | 3 | 8 | 9 | - |
| NLL | low | low | 10 | 7 | 13 | - |
| OLL | low | low | 10 | 10 | 7 | - |
| PLL | low | low | 11 | 2 | 4 | - |
| QLL | low | low | 10 | 7 | 8 | - |
| RLL | low | low | 10 | 8 | 7 | - |
| SLL | low | low | 10 | 7 | 3 | - |
| TLL | low | low | 11 | 3 | 2 | - |
| ULL | low | low | 8 | 8 | 8 | - |
| VLL | low | low | 10 | 4 | 7 | - |
| WLL | low | low | 10 | 9 | 7 | - |
| XLL | low | low | 10 | 5 | 6 | - |
| YLL | low | low | 10 | 11 | 8 | - |
| ZLL | low | low | 0 | 5 | 5 | - |
| ALH | low | high | 9 | 10 | 7 | 3 |
| BLH | low | high | 10 | 5 | 12 | 5 |
| CLH | low | high | 10 | 7 | 8 | 5 |
| DLH | low | high | 5 | 5 | 13 | 5 |
| ELH | low | high | 10 | 9 | 10 | 5 |
| FLH | low | high | 10 | 10 | 9 | 5 |
| GLH | low | high | 10 | 9 | 11 | 5 |
| HLH | low | high | 10 | 9 | 8 | 4 |
| ILH | low | high | 10 | 9 | 8 | 5 |
| KLH | low | high | 10 | 5 | 12 | 5 |
| LLH | low | high | 10 | 8 | 11 | 4 |
| MLH | low | high | 10 | 10 | 9 | 4 |
| NLH | low | high | 10 | 3 | 6 | 5 |
| OLH | low | high | 10 | 8 | 12 | 5 |
| PLH | low | high | 11 | 9 | 6 | 4 |
| QLH | low | high | 3 | 7 | 10 | 3 |
| RLH | low | high | 10 | 3 | 5 | 5 |
| SLH | low | high | 10 | 9 | 8 | 3 |
| TLH | low | high | 8 | 2 | 4 | 6 |
| ULH | low | high | 10 | 10 | 8 | 5 |
| VLH | low | high | 10 | 6 | 6 | 4 |
| WLH | low | high | 10 | 6 | 5 | 5 |
| XLH | low | high | 10 | 1 | 6 | 5 |
| YLH | low | high | 10 | 11 | 6 | 2 |
| ZLH | low | high | 10 | 0 | 0 |  |
| AHL | high | low | 11 | 6 | 9 | 5 |
| BHL | high | low | 10 | 8 | 11 | 5 |
| CHL | high | low | 10 | 8 | 10 | 4 |
| DHL | high | low | 8 | 5 | 4 | 4 |
| EHL | high | low | 3 | 1 | 2 | 3 |
| FHL | high | low | 7 | 8 | 8 | 5 |
| GHL | high | low | 3 | 8 | 11 | 4 |
| HHL | high | low | 3 | 8 | 8 | 5 |
| IHL | high | low | 10 | 9 | 9 | 4 |
| JHL | high | low | 10 | 12 | 8 | 5 |
| KHL | high | low | 4 | 11 | 5 | 5 |
| LHL | high | low | 10 | 4 | 8 | 4 |
| MHL | high | low | 5 | 4 | 12 | 4 |
| NHL | high | low | 10 | 0 | 0 |  |
| OHL | high | low | 10 | 1 | 2 | 3 |
| PHL | high | low | 10 | 6 | 9 | 5 |
| QHL | high | low | 8 | 5 | 12 | 4 |
| RHL | high | low | 10 | 11 | 3 | 2 |
| SHL | high | low | 10 | 3 | 4 | 4 |
| THL | high | low | 10 | 7 | 9 | 3 |
| UHL | high | low | 10 | 4 | 1 | 5 |
| VHL | high | low | 10 | 7 | 10 | 0 |
| WHL | high | low | 10 | 7 | 5 | 5 |
| XHL | high | low | 10 | 10 | 9 | 4 |
| YHL | high | low | 10 | 8 | 9 | 3 |
| ZHL | high | low | 10 | 7 | 3 | 3 |
| AHH | high | high | 11 | 5 | 4 | - |
| BHH | high | high | 10 | 7 | 10 | - |
| CHH | high | high | 10 | 9 | 9 | - |
| DHH | high | high | 10 | 4 | 6 | - |
| EHH | high | high | 10 | 11 | 7 | - |
| FHH | high | high | 3 | 6 | 8 | - |
| GHH | high | high | 10 | 9 | 9 | - |
| HHH | high | high | 10 | 10 | 6 | - |
| IHH | high | high | 3 | 4 | 10 | - |
| JHH | high | high | 10 | 0 | 0 | - |
| KHH | high | high | 3 | 10 | 8 | - |
| LHH | high | high | 10 | 11 | 8 | - |
| MHH | high | high | 9 | 12 | 3 | - |
| NHH | high | high | 11 | 5 | 3 | - |
| OHH | high | high | 10 | 6 | 8 | - |
| PHH | high | high | 10 | 3 | 3 | - |
| QHH | high | high | 10 | 8 | 7 | - |
| RHH | high | high | 10 | 4 | 5 | - |
| SHH | high | high | 10 | 4 | 0 | - |
| THH | high | high | 10 | 4 | 2 | - |
| UHH | high | high | 10 | 1 | 2 | - |
| VHH | high | high | 10 | 8 | 5 | - |
| XHH | high | high | 10 | 7 | 5 | - |
| YHH | high | high | 10 | 9 | 9 | - |
| ZHH | high | high | 10 | 8 | 9 | - |

**Table S2.** Characteristics of the six microsatellite loci used in this study. Characteristics were calculated from parental flies only in GenAlex, Genepop, or CERVUS. Table headings: N sample size, Na number of alleles, Ho observed heterozygosity, He expected heterozygosity, HWE evidence that locus is out of Hardy-Weinberg Equilibrium – ns not significant after Bonferroni correction.

| **Locus** | **N** | **Na** | **Ho** | **He** | **HWE** |
| --- | --- | --- | --- | --- | --- |
| **Tangus2** | 150 | 5 | 0.733 | 0.733 | ns |
| **Tangus8** | 155 | 6 | 0.716 | 0.742 | ns |
| **Tangus9** | 156 | 4 | 0.237 | 0.234 | ns |
| **Tangus10** | 143 | 6 | 0.657 | 0.726 | ns |
| **Tangus15** | 155 | 4 | 0.652 | 0.676 | ns |
| **Tangus20** | 156 | 6 | 0.750 | 0.786 | Ns |
| Combined non-exclusion probability (second parent):       0.026 | | | | | |

**Table S3**. Sample sizes in the differential allocation experiment, showing number of offspring of each sex that emerged as adults.

| **family** | **1^st^ male condition** | **housing treatment** | **# female offspring** | **# male offspring** |
| --- | --- | --- | --- | --- |
| LM1 | low | mating | 4 | 7 |
| LM2 | low | mating | 10 | 9 |
| LM3 | low | mating | 0 | 0 |
| LM4 | low | mating | 0 | 0 |
| LM5 | low | mating | 8 | 11 |
| LM7 | low | mating | 5 | 3 |
| LM8 | low | mating | 0 | 0 |
| LM9 | low | mating | 2 | 1 |
| LM10 | low | mating | 7 | 10 |
| LM11 | low | mating | 3 | 4 |
| LM12 | low | mating | 6 | 5 |
| LM13 | low | mating | 6 | 5 |
| LM14 | low | mating | 6 | 9 |
| LM15 | low | mating | 11 | 7 |
| LM16 | low | mating | 0 | 0 |
| LM17 | low | mating | 6 | 14 |
| LM18 | low | mating | 5 | 5 |
| LM19 | low | mating | 3 | 2 |
| LNM1 | low | no-mating | 5 | 4 |
| LNM2 | low | no-mating | 0 | 0 |
| LNM3 | low | no-mating | 1 | 4 |
| LNM4 | low | no-mating | 3 | 6 |
| LNM5 | low | no-mating | 7 | 8 |
| LNM6 | low | no-mating | 7 | 12 |
| LNM7 | low | no-mating | 2 | 3 |
| LNM8 | low | no-mating | 5 | 13 |
| LNM9 | low | no-mating | 7 | 5 |
| LNM10 | low | no-mating | 5 | 3 |
| LNM11 | low | no-mating | 0 | 0 |
| LNM12 | low | no-mating | 3 | 5 |
| LNM13 | low | no-mating | 1 | 3 |
| LNM14 | low | no-mating | 4 | 3 |
| LNM15 | low | no-mating | 3 | 7 |
| LNM16 | low | no-mating | 8 | 7 |
| LNM17 | low | no-mating | 4 | 6 |
| LNM18 | low | no-mating | 7 | 10 |
| LNM19 | low | no-mating | 4 | 5 |
| LP1 | low | partition | 8 | 9 |
| LP2 | low | partition | 11 | 7 |
| LP3 | low | partition | 5 | 4 |
| LP4 | low | partition | 0 | 0 |
| LP5 | low | partition | 0 | 0 |
| LP6 | low | partition | 11 | 8 |
| LP7 | low | partition | 0 | 0 |
| LP8 | low | partition | 12 | 7 |
| LP9 | low | partition | 2 | 2 |
| LP10 | low | partition | 2 | 8 |
| LP11 | low | partition | 0 | 2 |
| LP12 | low | partition | 6 | 7 |
| LP13 | low | partition | 1 | 5 |
| LP14 | low | partition | 2 | 0 |
| LP15 | low | partition | 5 | 8 |
| LP16 | low | partition | 0 | 0 |
| LP17 | low | partition | 3 | 2 |
| LP18 | low | partition | 5 | 7 |
| LP19 | low | partition | 11 | 9 |
| HM1 | high | mating | 0 | 0 |
| HM2 | high | mating | 0 | 5 |
| HM3 | high | mating | 3 | 1 |
| HM4 | high | mating | 0 | 0 |
| HM5 | high | mating | 10 | 9 |
| HM6 | high | mating | 2 | 4 |
| HM7 | high | mating | 4 | 2 |
| HM8 | high | mating | 7 | 11 |
| HM9 | high | mating | 2 | 0 |
| HM10 | high | mating | 2 | 3 |
| HM11 | high | mating | 12 | 3 |
| HM12 | high | mating | 3 | 1 |
| HM13 | high | mating | 5 | 5 |
| HM14 | high | mating | 7 | 7 |
| HM15 | high | mating | 6 | 8 |
| HM16 | high | mating | 7 | 3 |
| HM17 | high | mating | 6 | 5 |
| HM18 | high | mating | 9 | 8 |
| HNM1 | high | no-mating | 9 | 8 |
| HNM2 | high | no-mating | 5 | 5 |
| HNM3 | high | no-mating | 11 | 6 |
| HNM4 | high | no-mating | 8 | 9 |
| HNM5 | high | no-mating | 13 | 7 |
| HNM6 | high | no-mating | 4 | 7 |
| HNM7 | high | no-mating | 1 | 1 |
| HNM8 | high | no-mating | 0 | 0 |
| HNM9 | high | no-mating | 0 | 0 |
| HNM10 | high | no-mating | 12 | 6 |
| HNM11 | high | no-mating | 0 | 0 |
| HNM12 | high | no-mating | 0 | 5 |
| HNM13 | high | no-mating | 5 | 7 |
| HNM14 | high | no-mating | 9 | 7 |
| HNM15 | high | no-mating | 0 | 0 |
| HNM16 | high | no-mating | 6 | 5 |
| HNM17 | high | no-mating | 5 | 6 |
| HNM18 | high | no-mating | 7 | 7 |
| HNM19 | high | no-mating | 2 | 4 |
| HP1 | high | partition | 7 | 8 |
| HP2 | high | partition | 5 | 7 |
| HP3 | high | partition | 0 | 0 |
| HP4 | high | partition | 0 | 0 |
| HP5 | high | partition | 0 | 0 |
| HP6 | high | partition | 12 | 7 |
| HP7 | high | partition | 6 | 8 |
| HP8 | high | partition | 0 | 0 |
| HP9 | high | partition | 2 | 1 |
| HP10 | high | partition | 0 | 0 |
| HP11 | high | partition | 1 | 0 |
| HP12 | high | partition | 2 | 0 |
| HP13 | high | partition | 9 | 8 |
| HP14 | high | partition | 8 | 3 |
| HP15 | high | partition | 0 | 0 |
| HP16 | high | partition | 5 | 1 |
| HP18 | high | partition | 3 | 9 |
| HP19 | high | partition | 6 | 6 |

**Table S4**. Full models of effects of first and second male condition on offspring traits. Offspring body size (a) and egg size (b) were analysed using linear mixed models, with replicate (family) included as a random effect. The family variance component (proportion of total variance explained ± standard error) is shown below the fixed effects. Offspring egg-to-adult viability (c) and days to first emergence (d) were analysed using a generalized linear model with Poisson distribution and log link function.

|  | **Estimate** | **s.e.** | **d.f.** | **p** |
| --- | --- | --- | --- | --- |
| *(a) offspring body size* | | | | |
| first male condition | 0.230 | 0.090 | 1 | 0.012* |
| second male condition | 0.005 | 0.088 | 1 | 0.957 |
| first male × second male | 0.079 | 0.089 | 1 | 0.379 |
| offspring sex | -0.013 | 0.015 | 1 | 0.385 |
| first male × offspring sex | 0.007 | 0.015 | 1 | 0.632 |
| second male × offspring sex | -0.012 | 0.015 | 1 | 0.447 |
| 1^st^ male × 2^nd^ male × sex | 0.017 | 0.015 | 1 | 0.270 |
| maternal size | 0.204 | 0.095 | 1 | 0.036* |
| second male size | 0.023 | 0.095 | 1 | 0.805 |
| development time | 0.123 | 0.057 | 1 | 0.033* |
| family (random effect) = 0.700 ± 0.108 | | | | |
| *(b) egg size* |  |  |  |  |
| first male condition | 0.025 | 0.069 | 1 | 0.718 |
| second male condition | -0.086 | 0.069 | 1 | 0.220 |
| first male × second male | 0.037 | 0.069 | 1 | 0.596 |
| maternal size | 0.005 | 0.002 | 1 | 0.042* |
| first male × maternal size | 0.004 | 0.002 | 1 | 0.119 |
| second male × maternal size | 0.002 | 0.002 | 1 | 0.297 |
| 1^st^ male × 2^nd^ male × maternal | 0.001 | 0.002 | 1 | 0.741 |
| family (random effect) = 0.419 ± 0.071 | | | | |
| *(c) offspring egg-to-adult viability* | |  |  |  |
| first male condition | -0.063 | 0.036 | 1 | 0.074 |
| second male condition | -0.035 | 0.036 | 1 | 0.321 |
| first male × second male | 0.015 | 0.036 | 1 | 0.679 |
| egg size | -0.041 | 0.036 | 1 | 0.250 |
| maternal size | -0.141 | 0.035 | 1 | <.001* |
| *(d) offspring date of first emergence (developmental time)* | | | | |
| first male condition | -0.002 | 0.005 | 1 | 0.637 |
| second male condition | 0.002 | 0.005 | 1 | 0.761 |
| first male × second male | -0.003 | 0.005 | 1 | 0.599 |
| egg size | 0.002 | 0.005 | 1 | 0.646 |
| maternal size | 0.006 | 0.006 | 1 | 0.305 |
| # offspring emerged | -0.004 | 0.001 | 1 | 0.003* |
| *(e) offspring body size of genotyped individuals sired by 2^nd^ male* | | | | |
| treatment | 0.749 | 0.486 | 1 | 0.131 |
| maternal size | 1.243 | 0.662 | 1 | 0.068 |
| treatment × maternal size | 1.203 | 0.662 | 1 | 0.077 |
| paternal size | 0.504 | 0.493 | 1 | 0.312 |
| treatment × paternal size | 0.265 | 0.493 | 1 | 0.594 |
| maternal size × paternal size | 1.162 | 0.690 | 1 | 0.100 |
| treatment × maternal × paternal | 0.966 | 0.690 | 1 | 0.169 |
| sex | -0.024 | 0.182 | 1 | 0.897 |
| treatment × sex | 0.021 | 0.182 | 1 | 0.909 |
| maternal size × sex | -0.002 | 0.276 | 1 | 0.994 |
| treatment × maternal × sex | 0.092 | 0.276 | 1 | 0.740 |
| paternal size × sex | -0.020 | 0.191 | 1 | 0.915 |
| treatment × paternal × sex | 0.041 | 0.191 | 1 | 0.832 |
| maternal × paternal × sex | 0.131 | 0.298 | 1 | 0.661 |
| treatment × maternal × paternal × sex | 0.002 | 0.298 | 1 | 0.993 |
| family (random effect) = 0.706 ± 0.190 | | | | |

**Table S5. Effect of first male condition and housing treatment (mating, no mating, partition) on offspring body size. Effects were estimated in a linear mixed model. (a) Full model. (b) Partially simplified model re-fitted after removing interactions and covariates with p >0.2 in the full model. The family variance component (proportion of total variance explained ± standard error) is shown below the fixed effects for the partially simplified model.**

|  | Estimate | s.e. | d.f. | p |
| --- | --- | --- | --- | --- |
| *(a) Full model* |  |  |  |  |
| first male condition | 0.063 | 0.106 | 1 | 0.506 |
| housing treatment | 0.078 | 0.143 | 2 | 0.901 |
| first male condition × housing treatment | -0.295 | 0.147 | 2 | 0.099 |
| offspring sex | 0.027 | 0.014 | 1 | 0.146 |
| development time | -0.147 | 0.040 | 1 | <0.001* |
| housing treatment × development time | -0.042 | 0.047 | 2 | 0.109 |
| housing treatment × sex | 0.023 | 0.020 | 2 | 0.149 |
| first male condition × treatment × sex | -0.002 | 0.020 | 2 | 0.252 |
| first male condition × sex | 0.008 | 0.015 | 1 | 0.592 |
| maternal size | 0.055 | 0.111 | 1 | 0.622 |
| second male body size | 0.161 | 0.114 | 1 | 0.162 |
| *(b) Partially simplified model* |  |  |  |  |
| first male condition | 0.064 | 0.107 | 1 | 0.548 |
| housing treatment | 0.079 | 0.144 | 2 | 0.855 |
| first male condition × housing treatment | -0.297 | 0.147 | 2 | 0.087 |
| offspring sex | 0.024 | 0.014 | 1 | 0.088 |
| development time | -0.146 | 0.040 | 1 | <0.001* |
| housing treatment × development time | -0.043 | 0.047 | 2 | 0.088 |
| housing treatment × offspring sex | 0.029 | 0.020 | 2 | 0.124 |
| second male body size | 0.159 | 0.114 | 1 | 0.166 |
| family (random effect) = 0.825 ± 0.136 | | | | |

**Figure Legends**

**Figure S1.** Offspring were larger when the first male was in high condition, independent of the condition of the second male. Hollow points show family means, solid points show mean ± s.e. of family means.

**Figure S2.** (a) Offspring had reduced egg-to-adult viability when the first male was in high condition, but this effect was marginally non-significant when maternal body size was included in the model. Points show mean ± s.e. (b) Offspring viability was negatively related to maternal body size.

**Figure S3.** Observed number of individual offspring (a) and family groups (b) with zero allele mismatches to the second male far exceeds that which is expected from 10000 randomly drawn offspring-father pairs.
