# Supplementary figures and images for: Revisiting telegony: offspring inherit an acquired characteristic of their mother's previous mate
Source: Ecol Lett. 2014 Sep 30;17(12):1545–52. doi: 10.1111/ele.12373 (PMC4282758; doi:10.1111/ele.12373)

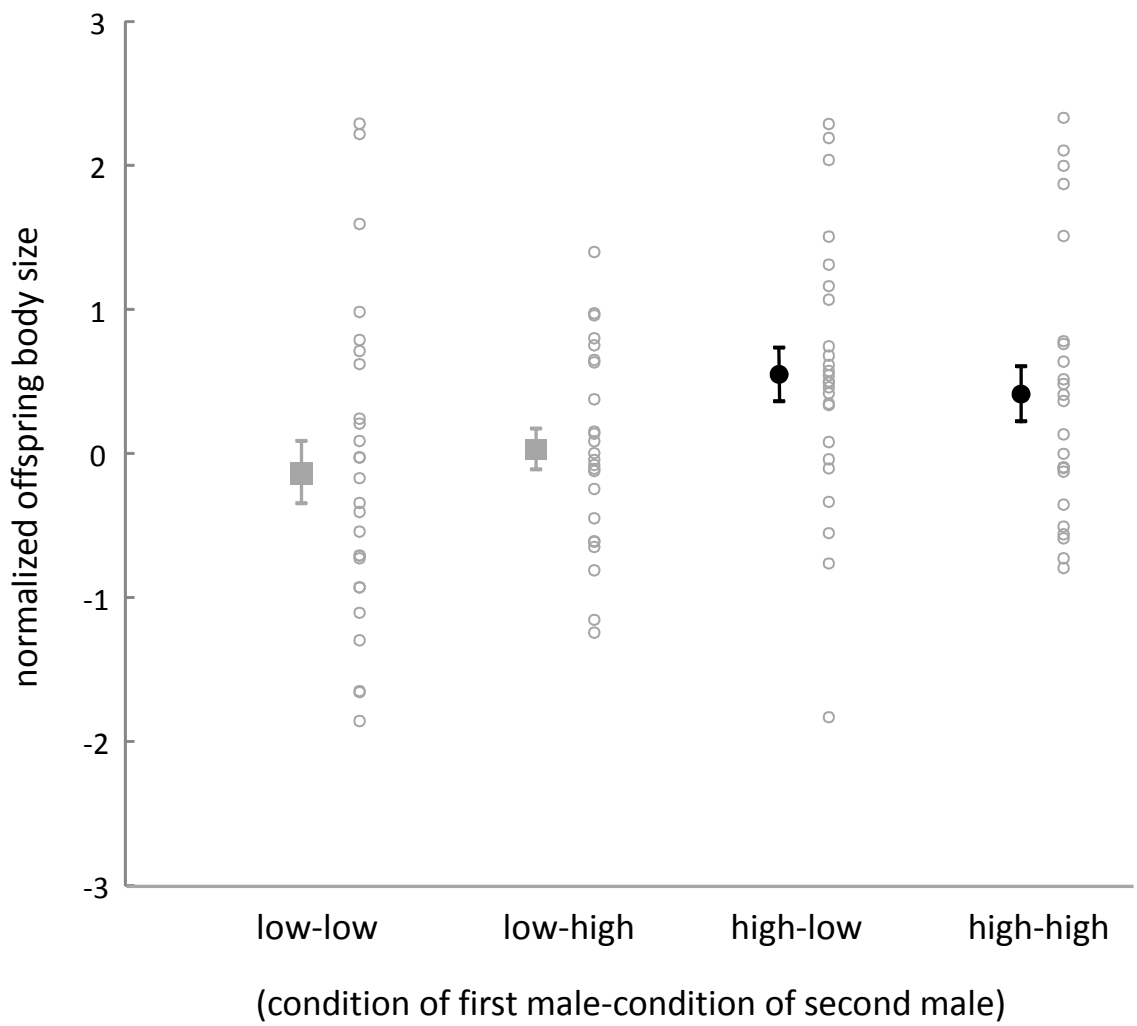

Supplement: Supplementary file 2 [file ele0017-1545-sd2.pdf]

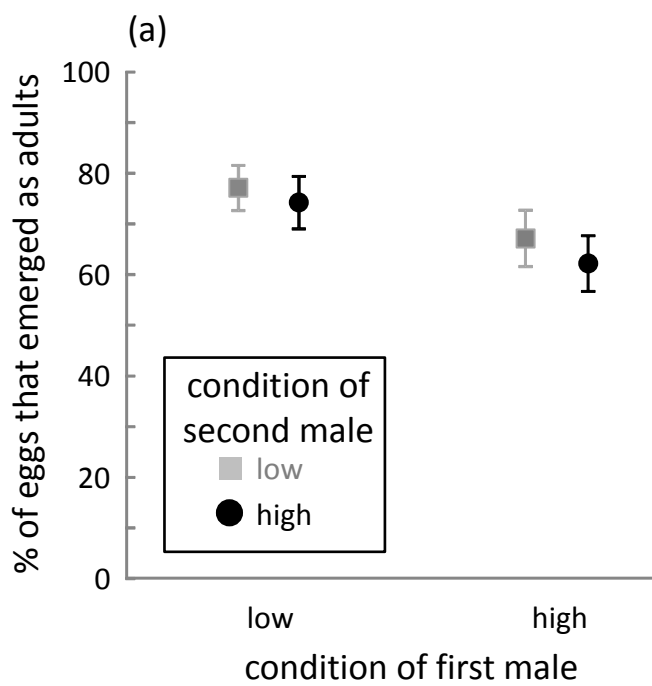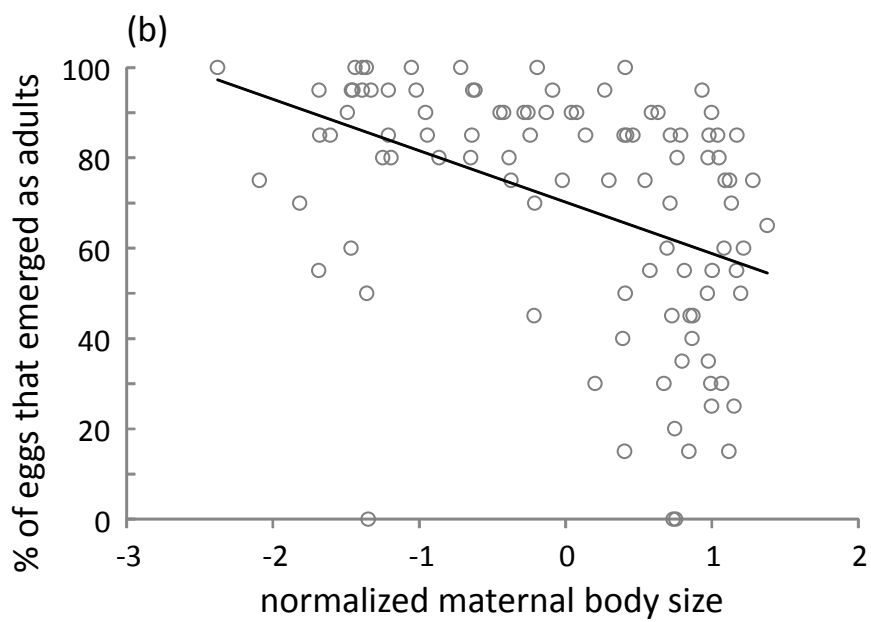

Supplement: Supplementary file 3 [file ele0017-1545-sd3.pdf]

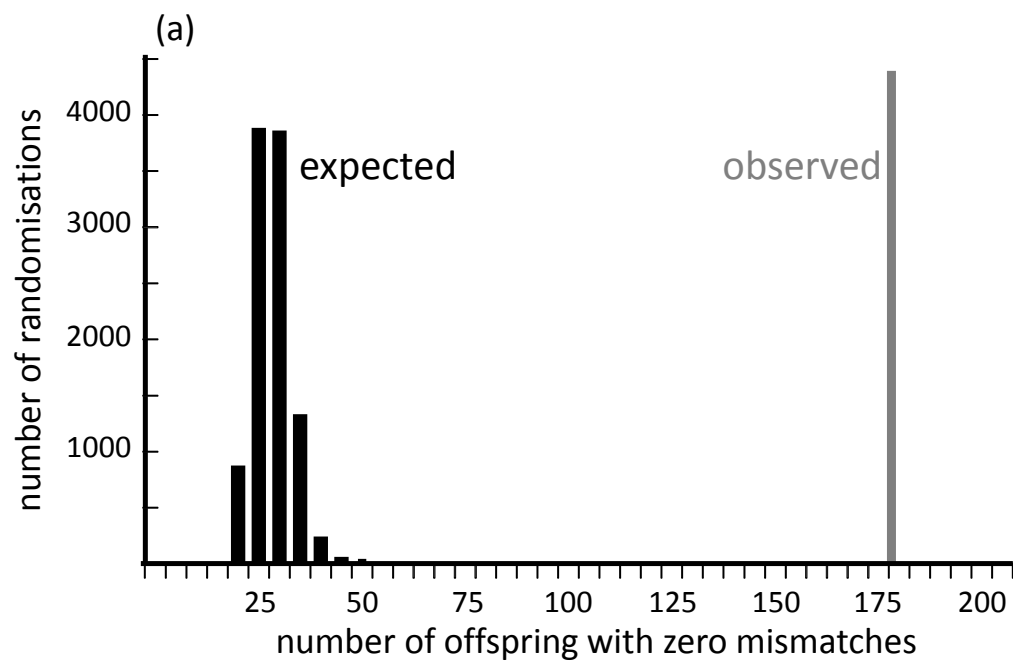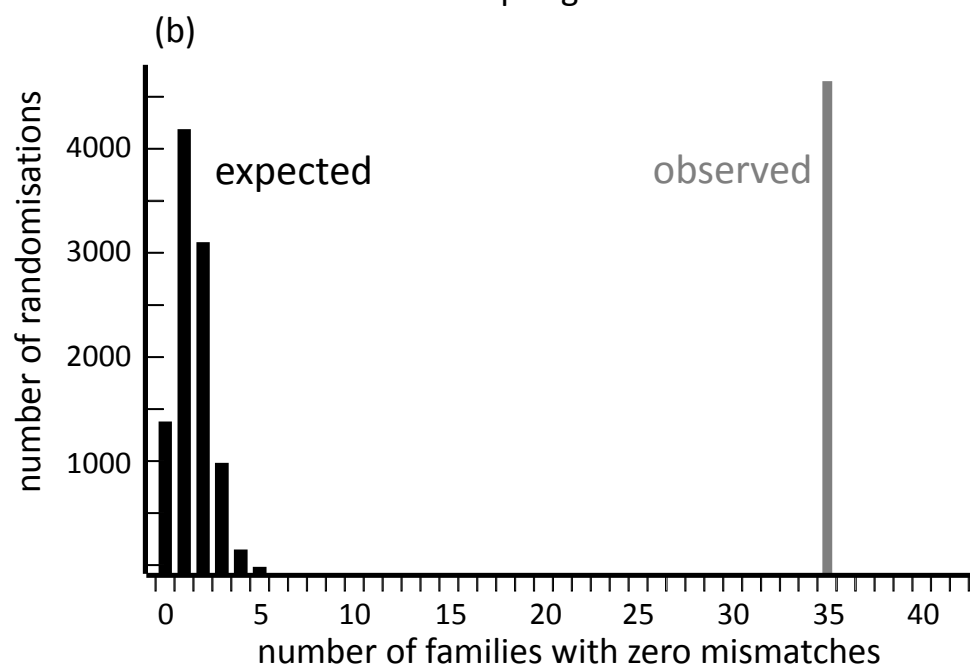

Supplement: Supplementary file 4 [file ele0017-1545-sd4.pdf]
